# Supplementary material for: Native-state proteomics of Parvalbumin interneurons identifies unique molecular signatures and vulnerabilities to early Alzheimer’s pathology
Source: Nat Commun. 2024 Apr 1;15:2823. doi: 10.1038/s41467-024-47028-7 (PMC10985119; doi:10.1038/s41467-024-47028-7)
Supplement: Supplementary file 1 — Supplementary Information [file 41467_2024_47028_MOESM1_ESM.pdf]

SUPPLEMENTARY INFORMATION

Native-state proteomics of Parvalbumin interneurons identifies unique molecular signatures and vulnerabilities to early Alzheimer's pathology

Prateek Kumar[#,1,2,3], Annie M Goettemoeller[#,2,4], Claudia Espinosa-Garcia[1,3], Brendan R. Tobin[5], Ali Tfaily[3], Ruth S. Nelson[3], Aditya Natu[1], Eric B. Dammer[2,6], Juliet V. Santiago[1,2,4], Sneha Malepati[2,7], Lihong Cheng[1,2], Hailian Xiao[1,2], Duc D. Duong[2,6], Nicholas T. Seyfried[1,2,6], Levi B. Wood[5,8],

Matthew JM Rowan[*,2,7], Srikant Rangaraju[*,1,2,3]

Affiliations:

1 Department of Neurology, Emory University School of Medicine, Atlanta, GA, 30322, USA

2 Center for Neurodegenerative Disease, Emory University School of Medicine

3 Department of Neurology, Yale University School of Medicine, New Haven, CT, 06510

4 Neuroscience Graduate Program, Laney Graduate School, Emory University

5 Georgia W. Woodruff School of Mechanical Engineering, Parker H. Petit Institute for Bioengineering and Bioscience, and Wallace H. Coulter Department of Biomedical Engineering, Georgia Institute of Technology, Atlanta, GA, 30322, USA

6 Department of Biochemistry, Emory University, Atlanta, GA, 30322, USA

7 Department of Cell Biology, Emory University School of Medicine, Atlanta, GA, 30322, USA

8 School of Chemical and Biological Engineering, Georgia Institute of Technology, Atlanta, GA, 30322, USA

#These authors contributed equally

*These authors jointly supervised this work

Corresponding author(s): mjrowan@emory.edu, srikant.rangaraju@yale.edu

Supplementary Figures: 11

Supplementary Data sheets: 7

Keywords: Interneurons, Proteomics, Biotinylation, Proximity labeling, Electrophysiology

Supplementary Figures and legends

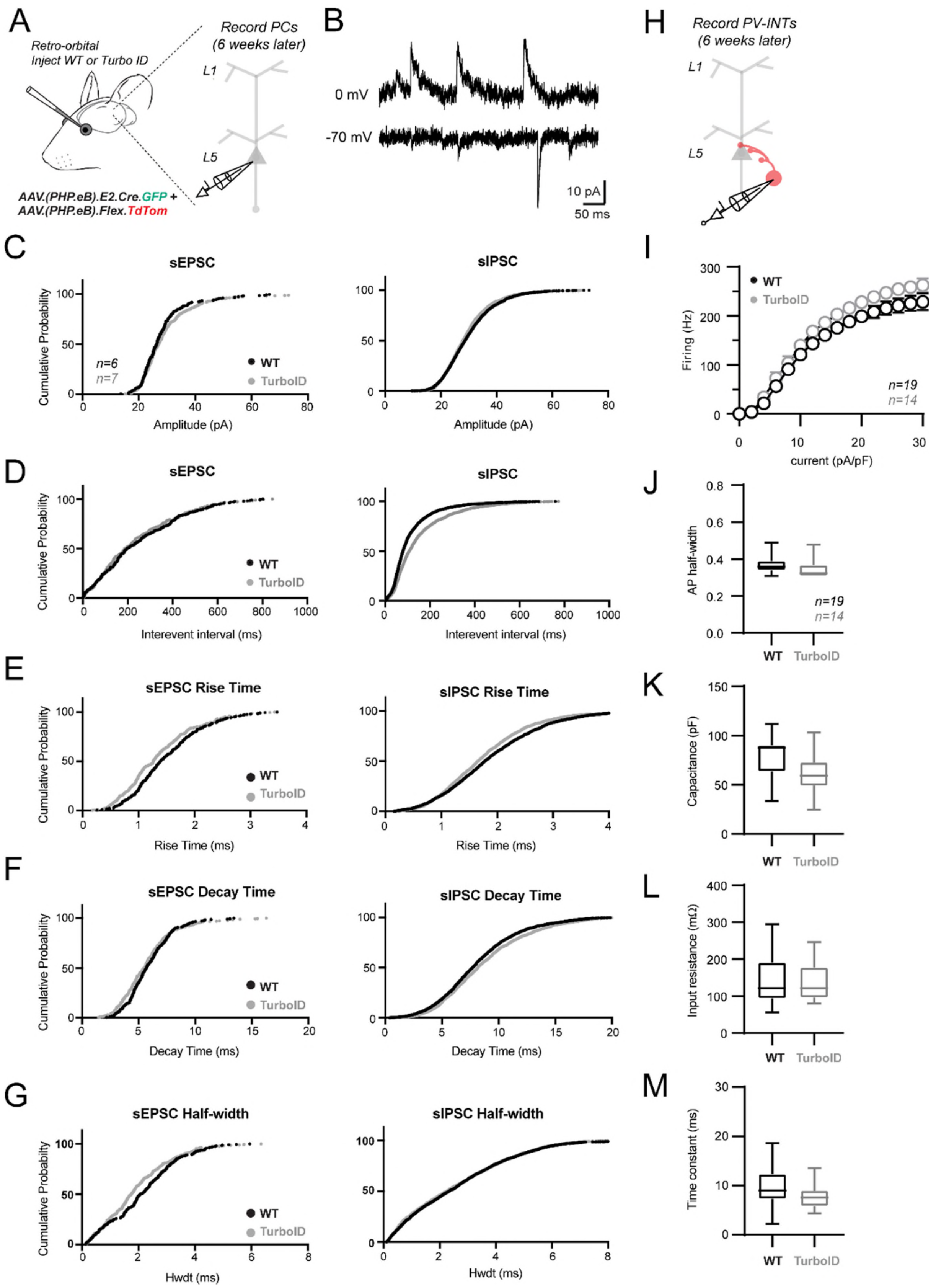

Supplementary Figure 1. PV-CIBOP does not disrupt PV-IN or local circuit properties

(related to Figure 1).

A. During procurement of CIBOP tissues from RO-injected Rosa26$^{TurboID/wt}$ (TurboID) and WT mice, a subset of the brain (SBFI region of S1 cortex) was used to immediately prepare acute slices for patch clamp recordings of unlabeled pyramidal neurons in layer 5.

B. Example traces from a voltage clamp recording in a pyramidal neuron in layer 5 cortex. -70 and 0 mV holding potentials were interleaved throughout the recording to sample spontaneous EPSCs and IPSCs, respectively.

C-G. Cumulative probability distribution curves for the amplitudes, frequency, and kinetic properties from all spontaneous EPSC and IPSC events recorded in pyramidal neurons of TurboID and WT mice.

H, I. In the same experiments as depicted in **A** and **B**, fluorescent-targeted current clamp recordings were performed in TdTomato+ neurons as identified using combined video-epifluorescent illumination. The GFP signal from the E2.Cre.GRP construct was not used, as it was generally much dimmer. Current injections (300 ms) of varying amplitude (0-30 pA/pF) were normalized to the individual cellular capacitance to control for potential variability between passive features.

J. Narrow action potential widths at half-maximal amplitude (half-width) quantified in TdTomato+ between WT and Turbo ID mice. Half-width was measured from the 1st spike elicited by current injection. Action potentials were generally ~0.35 ms, characteristic of fast-spiking cortical PV-INs.

K-M. Passive features measured in recordings from TdTomato+ PV-INs.

For synaptic properties recorded in pyramidal neurons in **C-G,** average values from all spontaneous events from individual recordings were used for statistical analysis comparing TurboID (n=7) and WT (n=6) mice. All comparisons were $p>0.05$, unpaired two-tailed T-test. For PV-IN recording data in **J-M**, all TurboID (n=19) and WT (n=14) comparisons were $p>0.05$, unpaired two-tailed T-test.

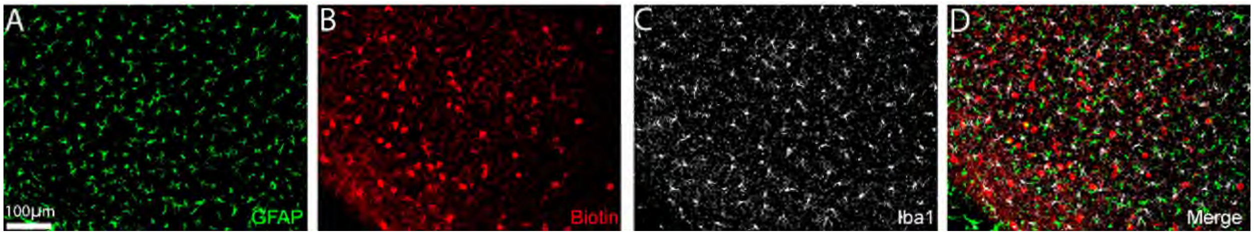

Supplementary Figure 2. PV-CIBOP specifically labels PV-INs without off-target labeling of astrocytes or microglia (related to Figure 1).

Representative immunofluorescence images (20x) from PV-CIBOP brain, confirming that biotinylation was not detected either in in astrocytes (GFAP+) or in microglia (Iba1+ positive). Furthermore, microglial and astrocyte morphology was qualitatively similar in labeled and non-labeled animals.

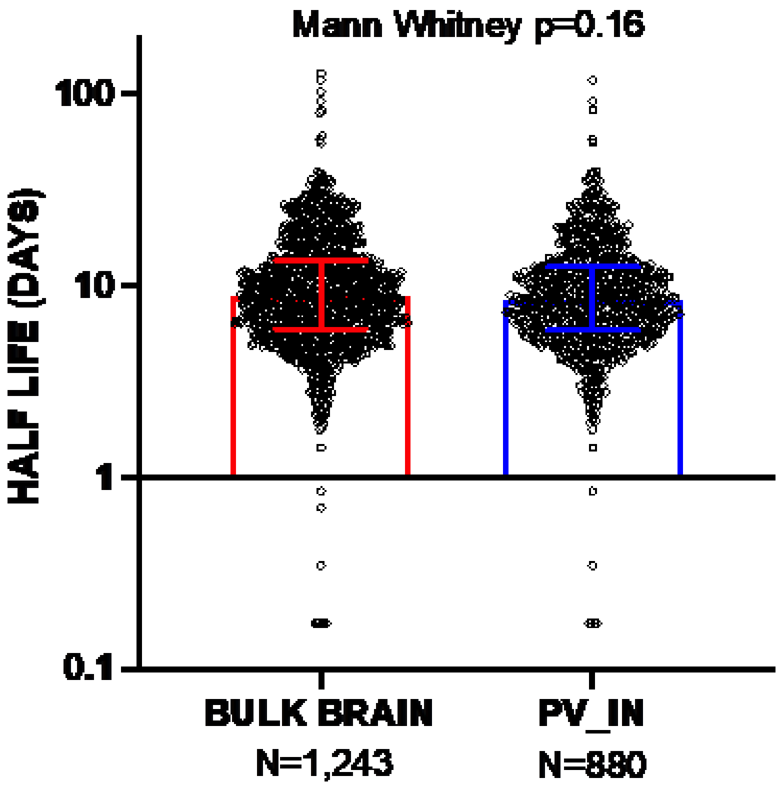

Supplementary Figure 3. PV-IN CIBOP-derived proteomes are not biased towards shorter or longer-lived proteins. Using brain-specific protein half-live estimates (in days) from Fornasiero et. al. Nature Communications 2018 (also see Supplementary Data 6), we compared half-life distributions of bulk brain proteins and PV-IN-enriched proteins from our CIBOP studies. The median half-life of proteins comparing PV-IN CIBOP proteomes and bulk brain proteomes were not statistically different, indicating that the CIBOP approach does not seem to be impacted by the rate of protein turnover (see Supplementary Data 1 sub-sheet 7 for source data related to protein half-life analysis).

Summary Table: Distribution of proteins based onRiboSeq_Neuropil vs. Soma (Glock et. al. PNAS 2021)

PV-IN proteins	Neuropil enriched	Soma enriched	Other	Total
N	64	397	181	642
%	9.97	61.84	28.19	100.00
**Bulk brain proteins**	Neuropil enriched	Soma enriched	Other	Total
N	95	520	265	880
%	10.80	59.09	30.11	100.00
**All 7,350 proteins (Glock et al.)**	Neuropil enriched	Soma enriched	Other	Total
N	807	2945	4098	7850
%	10.28	37.52	52.20	100.00

PV-IN vs. Bulk: Chi Square p = 0.56

Supplementary Figure 4. PV-IN CIBOP does not preferentially label neuronal proteins based on somatic or axon/synapse/dendritic localization. Comparison of distributions of soma-enriched, neuropil-enriched proteins in PV-IN proteomes, bulk brain proteomes as well as the reference Ribo-seq dataset by Glock et. al. PNAS 2021. Ribo-seq was used to measure mRNAs that were actively translated (translatome) in neurons, and classified as being enriched in somatic or neuropil (axon/dendrite/synapse) neuronal sub-compartments. Using these classifications of neuronal proteins, we assessed distributions of proteins identified in PV-INs by CIBOP, in the bulk brain proteomes from corresponding animals, and in the Glock et. al. reference Ribo-seq translatome. Chi-square statistics were used to compare distributions (see Supplementary Data 1 sub-sheet 6 related to protein localization analysis).

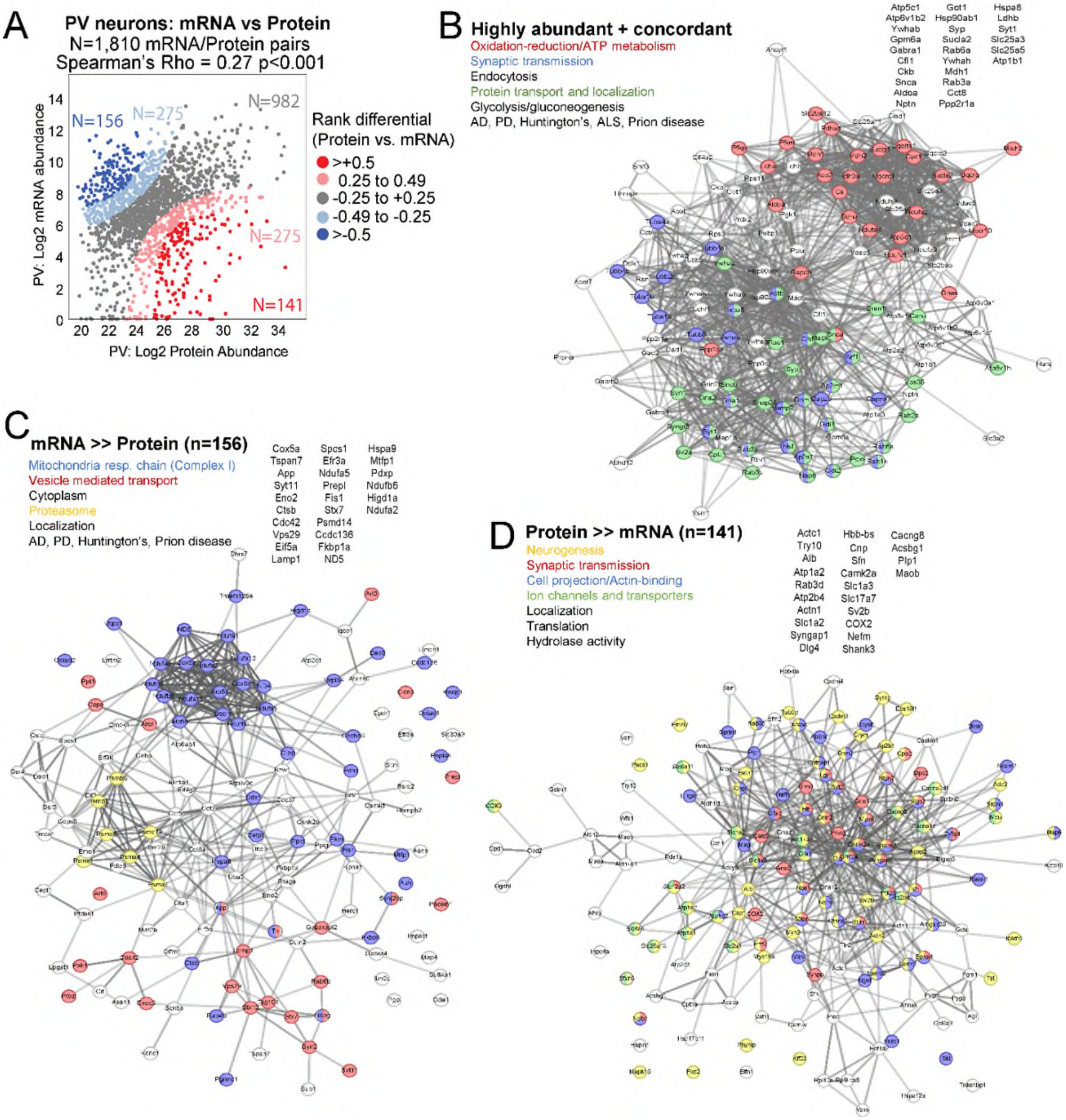

Supplementary Figure 5. Assessment of protein-mRNA discordance in PV-INs (related to Figure 1M).

A. Analysis of protein vs mRNA concordance in PV-INs, using PV-enriched proteins identified by PV-CIBOP and existing single nuclear transcriptomic data from the entire class of adult mouse PV-INs (Allen Brain Atlas). Based on differentials in rank abundances (protein vs. mRNA), discordant and concordant protein/mRNA pairs are highlighted. Spearman's Rho for protein vs. mRNA correlation is shown.

B-D. Three groups of protein/mRNA pairs were further analyzed: **B.** Group 1-Highly abundant and concordant: Protein rank differential between -0.25 and 0.49 and overall abundance >80th percentile in both protein and mRNA datasets). **C.** Group 2: Discordance with mRNA greater than protein abundance (Protein vs. mRNA rank differential < -0.5). **D.** Group 3: Discordance with protein greater than mRNA abundance (Protein vs. mRNA rank differential > +0.5). Top gene symbols/protein IDs, top GSEA terms representative of each group, and STRING PPI diagrams (with colors indicating each ontology), are shown in each panel. Higher mRNA/protein concordance was observed in ontologies including oxidative-reduction processes, ATP generation, synaptic transmission, glucose metabolism, endocytosis and protein transport. Low abundance proteins with high mRNA abundance were enriched in ontologies related to complex I respiratory chain, proteasome, cytosolic and vesicle-mediated transport terms. Proteins with high abundance but low mRNA expression were enriched in neurogenesis, ion channel function and transporters, cytoskeletal/cell projection and translation-related proteins. Please see Supplementary Data 1 sub-sheet 5 for related source data and analyses.

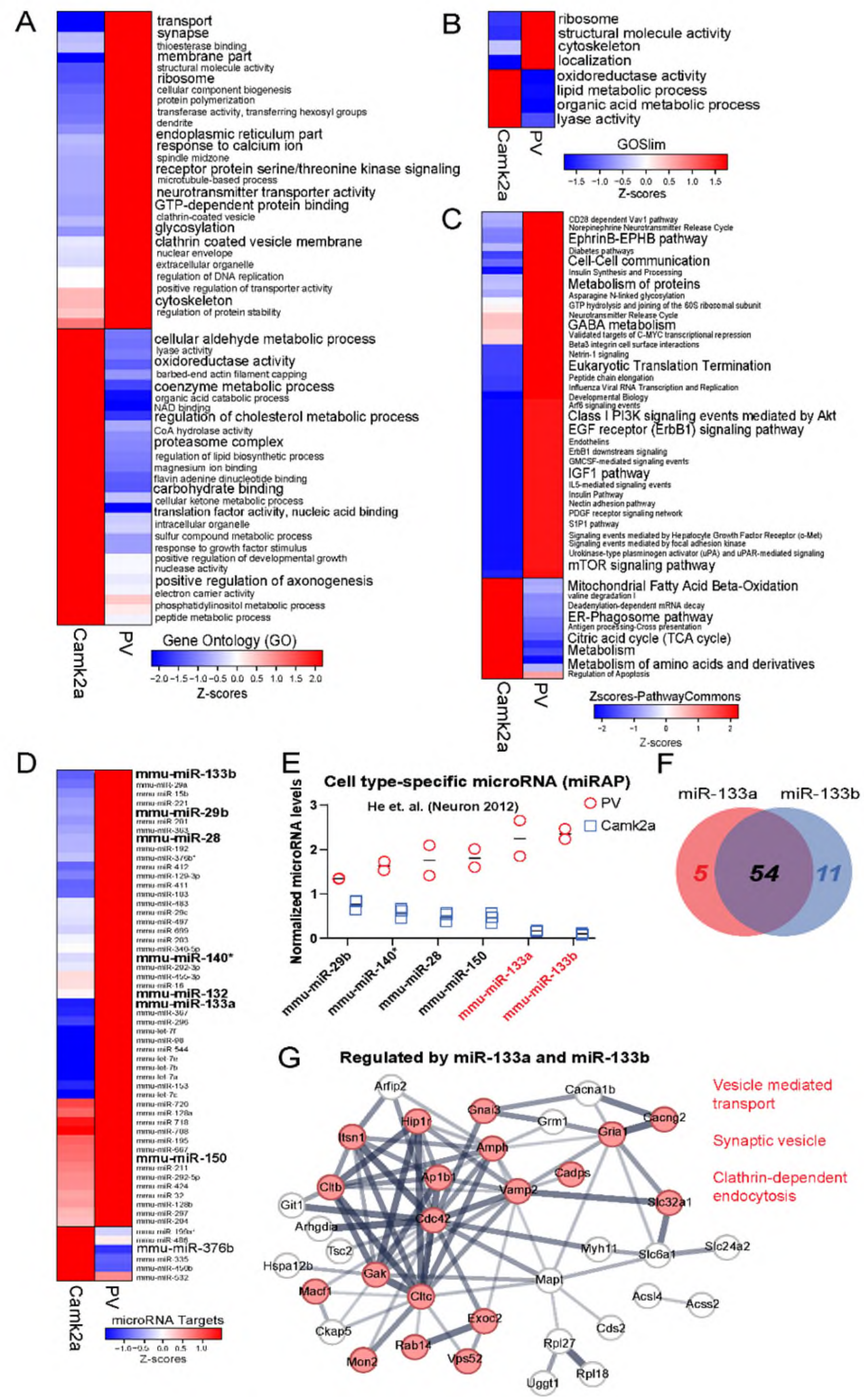

Supplementary Figure 6. GSEA of PV-IN and Camk2a neuronal CIBOP proteomes (related to Figure 2).

A-D. Heatmap representation of (**A**) Gene Ontology (GO) terms, (**B**) GOSlim terms, (**C**) PathwayCommons terms as well as (**D**) predicted upstream microRNA (miRNA) regulators that are over-represented in proteomic signatures of PV-INs and Camk2a neurons, using the CIBOP approach.

E. Normalized miRNA levels as measured using the miRAP method (He et. al. Neuron 2012) that identified miRNAs that were differentially abundant in PV-INs and Camk2a neurons. The miRNAs enriched in PV-INs that are represented in this graph (particularly miR-133a and miR-133b), were also predicted as up-stream regulators of PV-IN proteomic signatures. Therefore, miR-133a and miR-133b are highly likely miRNA regulators that may be functionally important regulators of PV-INs.

F. Venn Diagram representing shared and distinct target genes that are regulated by miR-133a and miR133-b in PV-IN proteomes.

G. 54 proteins were identified as shared down-stream targets of both miR-133a and miR-133b in PV-INs, and these are represented as a STRING PPI network. Top GO terms emerging from this analysis include synaptic vesicle, vesicle transport and clathrin-dependent endocytosis terms. This indicates that miR-133a and miR-133b may be regulators of synaptic vesicle related function in PV-INs. Also see Supplementary Data 2 for related analyses.

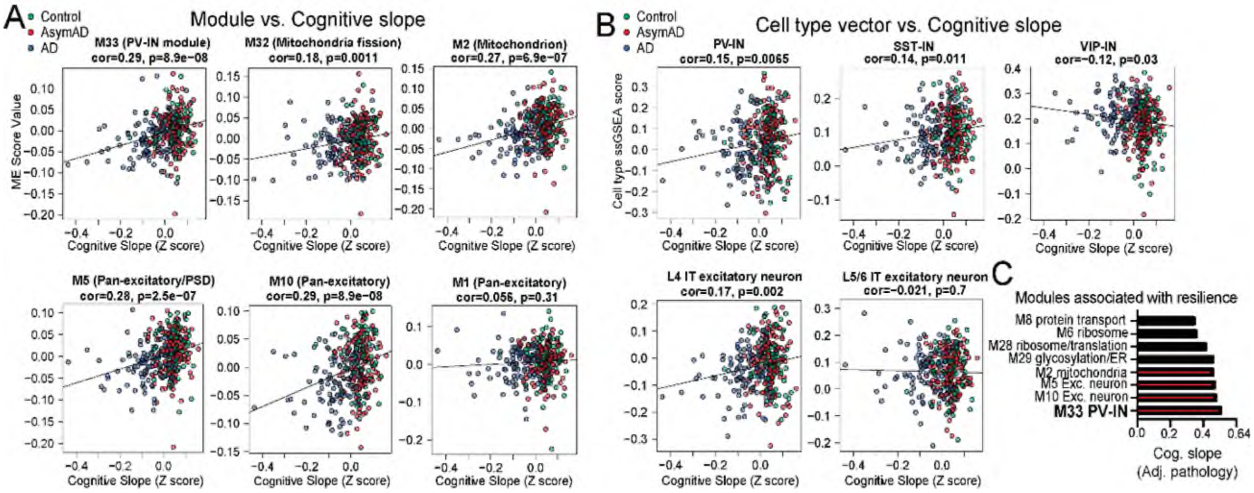

Supplementary Figure 7. Associations between brain protein co-expression modules and cell type abundance vectors with cognitive slope in humans (related to Figure 3).

A, B. Associations of module eigenprotein (ME) (**A**) and cell type vectors (**B**) with cognitive slope for modules with highest positive correlations with cognitive slope. A higher cognitive slope indicates cognitive stability (or resilience) while a lower (negative) slope indicates faster cognitive decline over time. Color (reg, light blue and dark blue represent controls, AsymAD and AD cases respectively).

C. Module eigenprotein associations with cognitive slope (resiliency) after adjustment for neuropathological features. This shows that module M33, followed by excitatory neuron modules (M5, M10) and mitochondrial modules (M2) had the highest correlation with cognitive resilience.

See Supplementary Data 3 for related analyses and source data.

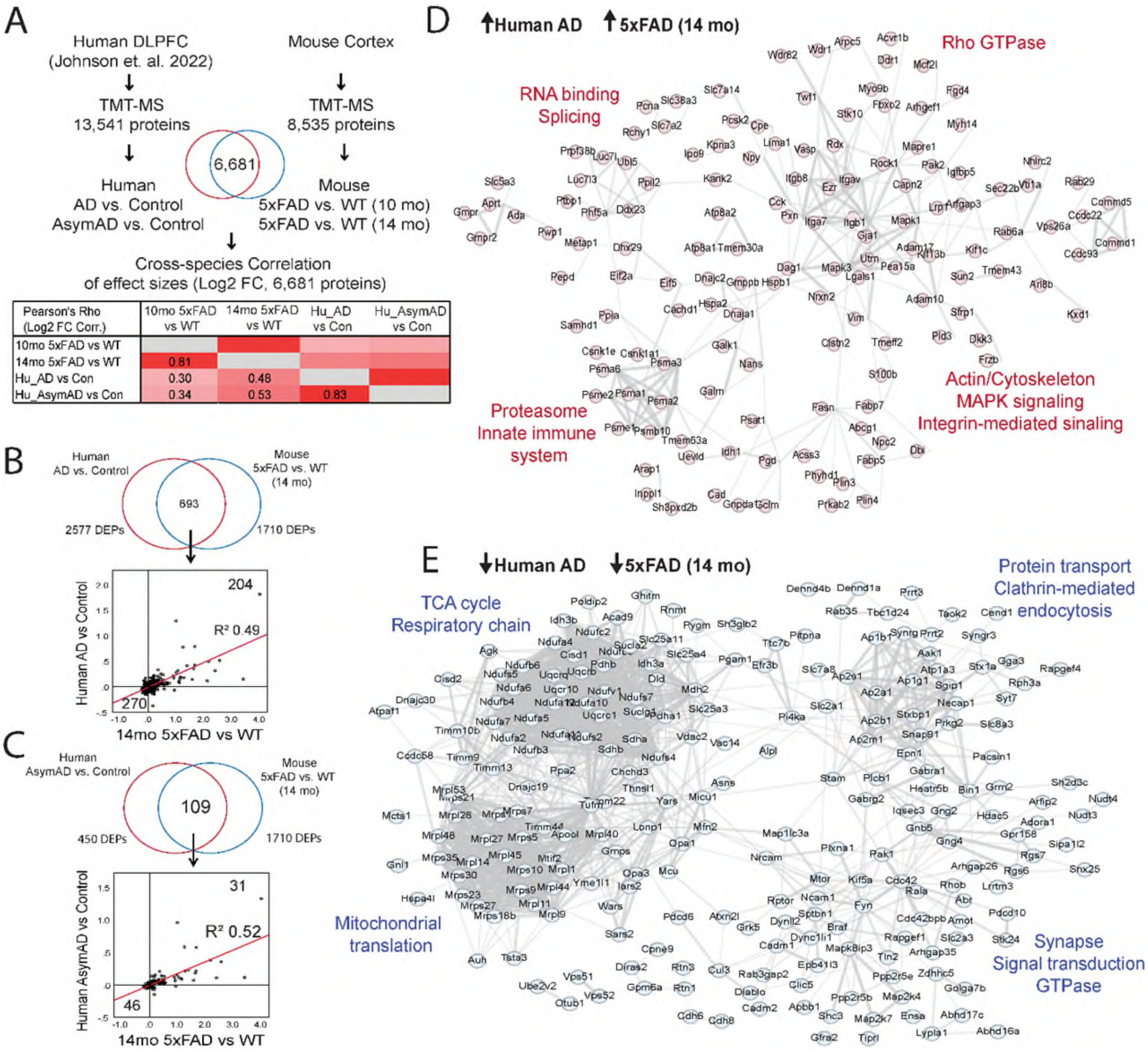

Supplementary Figure 8. Analysis of concordance between human AD and mouse 5xFAD pathological changes using MS-based proteomics.

A. Post-mortem brain proteomic data were obtained from Johnson et. al. (Nat. Neuroscience 2022) in which >13,500 proteins were quantified by TMT-MS. This dataset was derived from post-mortem dorsolateral pre-frontal cortex from subjects with AD+dementia (AD), asymptomatic AD (AsymAD) and from controls (no evidence of AD or other neurodegenerative disease by pathology). Of these, 6,681 proteins were also quantified in our mouse bulk proteomic TMT-MS dataset (also see **Supplementary Data 4**). Pearson's correlation coefficients were calculated, correlating log2-transformed fold changes (Log2FC) (Humans: AD vs. Control, AsymAD vs. Control; Mice: 14 mo 5xFAD vs. Control; 10 mo 5xFAD vs. Control). Pearson's was appropriate given the normal distribution and continuous nature of log2-transformed FC data. These were summarized as a heat-map (deep red indicating a Pearson's Rho =1, 0 indicating Rho =0).

B. Log2FC correlations between humans (AD vs. Control) and mice (14 mo 5xFAD vs. WT) limited to DEPs in both datasets. Pearson R2 values are shown. Number is each quadrant represent number of proteins.

C. Log2FC correlations between humans (AsymAD vs. Control) and mice (14 mo 5xFAD vs. WT) limited to DEPs in both datasets. Pearson R2 values are shown. Number is each quadrant represent number of proteins.

D, E. STRING protein-protein-interaction networks highlighting pathways/mechanisms that are conserved across both species. Panel **D** represents concordant proteins and pathways that are increased in both human AD and in 5xFAD mice at 14 months of age. Panel **E** represents concordant proteins and pathways decreased in both human AD and 5xFAD mice at 14 months of age.

Please see Supplementary Data 4 sub-sheet 4 for related source data and analyses.

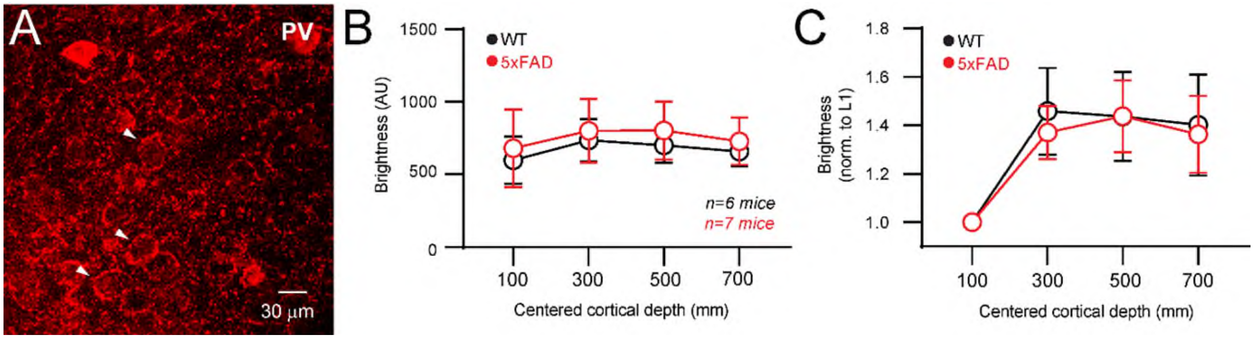

Supplementary Figure 9. Parvalbumin IHC in WT and 5xFAD mice (related to Figure 4D-G).

A. Representative IHC image of anti-parvalbumin staining in the L5 region of S1 cortex. White arrows denote examples of basket structures surrounding putative pyramidal and other neuron types. Extensive staining of PV-IN presynaptic basket structures was evident using this antibody staining method.

B. Quantification of integrated fluorescence (in arbitrary units) from Alexa-594 secondary directed against Parvalbumin. Z-stacks were obtained from thin slices cortical WT and 5xFAD tissues. An ~200 μm FOV 60X objective allowed for images centered at 100, 300, 500, and 700 (± 100) micron cortical depths. Background fluorescence measured at an offset location was subtracted from all images.

C. Same quantification as in (B) but normalized to the FOV centered at 100 μm cortical depth. Reduced expression was apparent at this superficial depth, likely due to lack of extensive Parvalbumin-labeled structures with respect to deeper layers. Staining was consistent across all deeper areas corresponding to Layers 2/3, 4, and 5 and did not differ between WT and 5xFAD cortices.

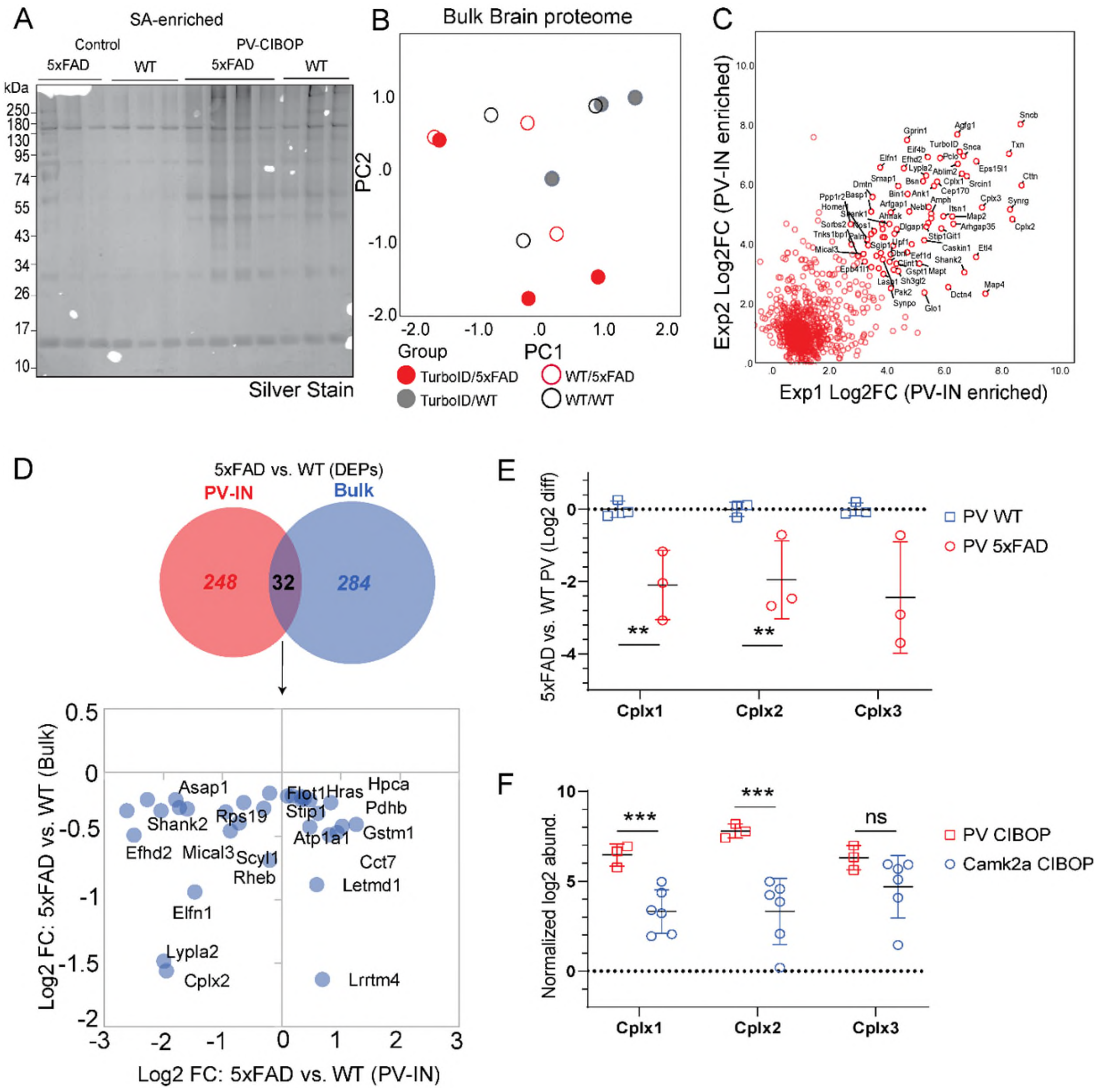

Supplementary Figure 10. PV-CIBOP identifies proteomic changes occurring in PV-INs in early stages of Aβ pathology (related to Figure 5).

A. Silver-stained gel of SA-enriched PV-IN proteins from experimental animals (in Figure 5) confirming higher protein enrichment from PV-CIBOP animals as compared to control animals.

B. PCA of bulk brain (input) proteomes showing lack of an observable group-based clustering based on genotype (WT vs 5xFAD) or biotinylation (CIBOP vs controls). This contrasts with genotype differences observed in PV-IN proteomes presented in Figure 5 (see Source data file for PCA results).

C. Level of agreement between two independent PV-IN proteomes using the CIBOP approach. Experiment 1: PV-CIBOP in WT mice presented in Figure 1. Experiment 2: PV-CIBOP in WT mice presented in Figure 5. Log2 fold changes (CIBOP vs control) of proteins that were labeled

in both datasets, are shown. Overall correlation between two studies was moderate (Pearson's Rho =0.61, $p<0.001$). Top PV-IN proteins identified by both studies, were similar (including Snca, Sncb, Cplx1, Cplx2, Cplx3, Elfn1, Bsn, as well as TurboID).

D. DEPs (comparing 5xFAD vs. WT) identified at the level of the bulk proteome and PV-IN proteome were distinct except for 32 proteins (intersect). Agreement in level of differential abundances (log2 FC 5xFAD vs WT) between bulk and PV-IN proteomes was modest with the exception of some proteins (eg. Cplx2, Lypla2, Elfn1) which shown concordant decreased levels in both bulk and PV-IN proteomes.

E. Protein levels of three complexins in the PV-IN proteome, comparing 5xFAD vs. WT genotypes (*$p<0.05$, **$p<0.01$, ***$p<0.005$).

F. Protein levels of three complexins in PV-IN vs. Camk2a CIBOP proteomes (*$p<0.05$, **$p<0.01$, ***$p<0.005$).

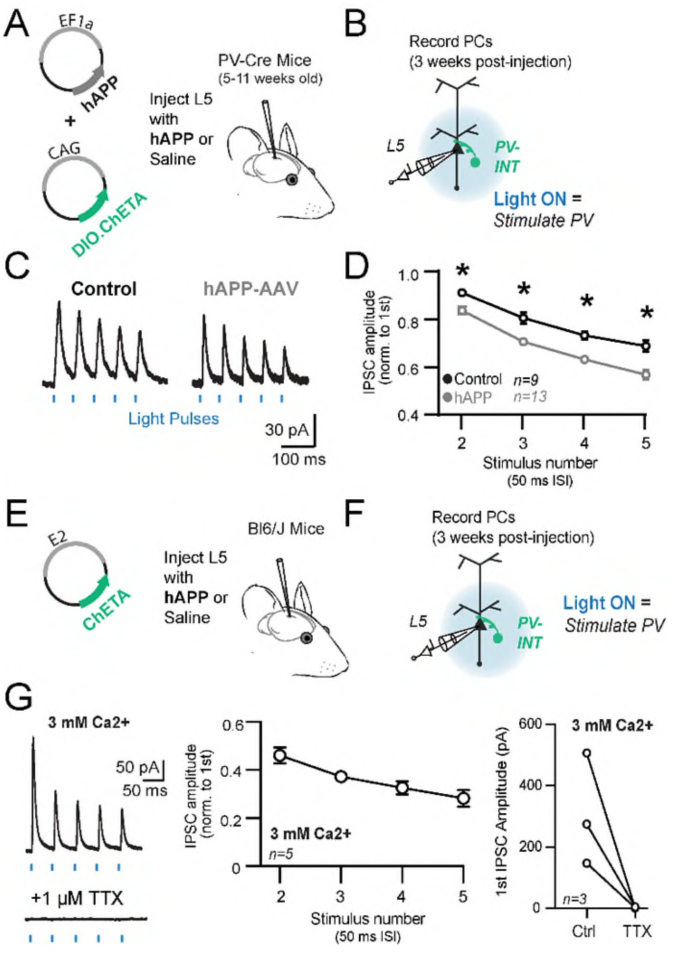

Supplementary Figure 11. hAPP-AAV effect on PV neurotransmission (related to Figure 6).

A. Experimental outline: PV-Cre mice were injected with AAV.CAG.DIO.ChETA and either with or without AAV.EF1a.hAPP in the somatosensory cortex at 5-11 weeks of age.

B. Three weeks post-injection, PV interneurons were stimulated at 20 Hz and nearby pyramidal cells were patched to examine the PV-PC paired-pulse ratio (PPR) and the multiple-pulse ratio (MPR). For **B-D**, 1.5mM external Ca^{2+} was used.

C. Example traces of optogenetically-evoked PV inhibitory post-synaptic currents on pyramidal cells for saline-injected (left) and hAPP-AAV injected (right) cortices using 1.5mM external Ca^{2+}.

D. IPSCs in AAV-hAPP injected mice displayed a significant change in synaptic dynamics as measured using MPR across all measured stimuli at 20 Hz. (*$p<0.05$ Two-way ANOVA with Sidak's posthoc comparisons for each stimulus between hAPP and saline control experiments).

E. Experimental outline: AAV.E2.ChETA was injected in WT mice in the somatosensory cortex.

F. Three weeks post-injection, as depicted in panel **E**, PV interneurons were stimulated with blue light pulses at 20 Hz and nearby pyramidal cells were patched to examine the PV-PC PPR and MPR at 3mM external Ca^{2+}.

G. AAV.E2.ChETA-driven IPSCs were strongly depressing with 3mM external Ca^{2+}. IPSCs in these conditions were completely abolished after the application of 1μM TTX in the bath solution.
