# Supplementary material for: Native-state proteomics of Parvalbumin interneurons identifies unique molecular signatures and vulnerabilities to early Alzheimer’s pathology
Source: Nat Commun. 2024 Apr 1;15:2823. doi: 10.1038/s41467-024-47028-7 (PMC10985119; doi:10.1038/s41467-024-47028-7)
Supplement: Supplementary file 3 — Description of Additional Supplementary Files [file 41467_2024_47028_MOESM3_ESM.pdf]

Description of Additional Supplementary Files

File Name: Supplementary Data 1
Description: LFQ-MS data related to Figure 1: PV-IN proteome.

File Name: Supplementary Data 2
Description: LFQ-MS data related to Figure 2: PV-IN vs Camk2a CIBOP proteome.

File Name: Supplementary Data 3
Description: Neuronal subtype marker lists used for analyses in Figure 3.

File Name: Supplementary Data 4
Description: Mouse TMT-MS data related to Figure 4.

File Name: Supplementary Data 5
Description: PV-CIBOP in 3 mo 5xFAD and WT mice: Bulk and SA-enriched proteomes and associated analyses, related to Figure 5.

File Name: Supplementary Data 6
Description: Analysis of protein half-life distribution in PV-IN DEPs (5xFAD vs. WT CIBOP), related to Figure 7.

File Name: Supplementary Data 7
Description: List of reagents including antibodies.
