# Supplementary material for: Native-state proteomics of Parvalbumin interneurons identifies unique molecular signatures and vulnerabilities to early Alzheimer’s pathology
Source: Nat Commun. 2024 Apr 1;15:2823. doi: 10.1038/s41467-024-47028-7 (PMC10985119; doi:10.1038/s41467-024-47028-7)
Supplement: Supplementary file 11 — Reporting Summary [file 41467_2024_47028_MOESM11_ESM.pdf]

nature portfolio

Corresponding author(s): Srikant Rangaraju
Last updated by author(s): Mar 8, 2024

Reporting Summary

Nature Portfolio wishes to improve the reproducibility of the work that we publish. This form provides structure for consistency and transparency in reporting. For further information on Nature Portfolio policies, see our Editorial Policies and the Editorial Policy Checklist.

Statistics

For all statistical analyses, confirm that the following items are present in the figure legend, table legend, main text, or Methods section.

n/a	Confirmed	
☐	☒	The exact sample size (*n*) for each experimental group/condition, given as a discrete number and unit of measurement
☐	☒	A statement on whether measurements were taken from distinct samples or whether the same sample was measured repeatedly
☐	☒	The statistical test(s) used AND whether they are one- or two-sided<br>*Only common tests should be described solely by name; describe more complex techniques in the Methods section.*
☐	☒	A description of all covariates tested
☐	☒	A description of any assumptions or corrections, such as tests of normality and adjustment for multiple comparisons
☐	☒	A full description of the statistical parameters including central tendency (e.g. means) or other basic estimates (e.g. regression coefficient) AND variation (e.g. standard deviation) or associated estimates of uncertainty (e.g. confidence intervals)
☐	☒	For null hypothesis testing, the test statistic (e.g. *F*, *t*, *r*) with confidence intervals, effect sizes, degrees of freedom and *P* value noted<br>*Give P values as exact values whenever suitable.*
☒	☐	For Bayesian analysis, information on the choice of priors and Markov chain Monte Carlo settings
☐	☒	For hierarchical and complex designs, identification of the appropriate level for tests and full reporting of outcomes
☐	☒	Estimates of effect sizes (e.g. Cohen's *d*, Pearson's *r*), indicating how they were calculated

Our web collection on statistics for biologists contains articles on many of the points above.

Software and code

Policy information about availability of computer code

Data collection

Clampfit (Molecular Devices) for electrophysiology recording
Keyence BZ-X810
Keyence BZ-X810 Analyzer
Odyssey Infrared Imaging System: LI-COR Biosciences
ChemiDoc Imaging System (Bio-Rad)
BD LSR II flow cytometer
Gen5™ Microplate ReaderGen5™ Microplate Reader
MAGPIX instrument (Luminex)
Orbitrap Lumos Tribrid mass spectrometer

Data analysis

Single nucleus Allen brain atlas snRNA data was downloaded from https://cells.ucsc.edu/?ds=allen-celltypes+human-cortex+various-corticalareas&+meta=class_label and processed in R to generate a count per million normalized reference matrix with 47,509 non-excluded cell nuclei assigned to any of 19 cell type clusters. The 598 sample Banner + ROSMAP consensus proteome protein profiles of the bulk dorsolateral prefrontal cortex (BA-9) from postmortem human donors was the bulk brain data for deconvolution, or ultimately, across-sample, within cell type relative abundance estimation48. EnsDeconv was run with some adjustments per: https://randel.github.io/EnsDeconv/reference/get_params.html and https://randel.github.io/EnsDeconv/. Briefly, the 5 marker identification methods used to get the top 50 markers by each method were t, wilcox, combined, "none" (i.e., all genes in the snRNA reference as a profile), and regression. All methods were run for both untransformed and log2-transformed data. CIBERSORT was used as the most efficient deconvolution method with a low profile for RAM use and CPU time, and estimates from 9 of 10 successful combinations of the above marker selection and transformation

methods with CIBERSORT estimation. The nine individual marker selection methods produced a redundant total of 350 marker genes, and of these, genes present in all 9 of the lists for each respective cell type were kept as a consensus list of markers. These consensus lists (Supplemental Datasheet 3) were used as input into the GSVA R package implementation of the ssGSEA algorithm49. Finally, ssGSEA estimates of within-cell type relative abundances across the 488/598 samples in the published consensus protein network48 were correlated to the 44 module eigenproteins (MEs), which are the first principal components of each module in the network, in addition to the ROSMAP cohort-specific trait of the slope of cognitive decline, a Z score-scaled measure indicating degree of cognitive resilience of an individual compared to the mean age-dependent cognitive decline of the full ROSMAP cohort population34,35. Correlation was performed using the WGCNA R package (vl.72-1) function plotEigengeneNetworks. For resilience, PWAS enrichment of significance among PV-IN or CAMK2A neuron-enriched protein gene products (Fig. 2F), and for PV-IN 5xFAD DEPs (Fig. 50), permutation-based enrichment of pooled significance from the PWAS was computed as previously published ()36, Software for this is available from https://www.github.com/edammer/MAGMA.SPA.
Other sources of data used for analyses in this manuscript MicroRNA affinity purification (miRAP) data from studies of PV-IN and Camk2a neurons was downloaded from supplemental information associated with the original miRAP publication30 and miRNA species with PV-IN vs. Camk2a neuronal enrichment patterns, were cross-referenced with predicted miRNA regulators in our PV-CIBOP and Camk2a-CIBOP studies. Protein-protein-interactions between proteins within lists of interest were examined using STRING (https://stringdb.org/cgi/input?sessionId=bqsnbjruDXP6&input_page_show_search=on). The reference gene sets for GSVA were the MS (Mouse) Ontology Gene Sets from MSigDB (https://www.gseamsigdb. org/gsea/msigdb/mouse/collections.jsp?targetSpeciesDB=Mouse#MS).
Within biotinylated proteins, group comparisons were performed using a combination of approaches, including differential abundance analysis, hierarchical clustering analysis (Broad Institute, Morpheus, https://software.broadinstitute.org/morpheus), as well as PCA, (in SPSS Ver 26.0 or R). A complete description of the TMT mass spectrometry study, including methods for sample preparation, mass spectrometry was not certified by peer review) is the author/funder. All rights reserved. No reuse allowed without permission. bioRxiv preprint doi: https://doi.org/10.1101/2023.05.17.541038; this version posted May 17, 2023. The copyright holder for this preprint (which 40 methodology and data processing, are available online (https://www.synapse.org/#!Synapse:syn27023828). Mass spectrometry raw data were processed in Proteome Discover (Ver 2.1) and then searched against Uniprot mouse database (version 2020), and then processed downstream as described for human brain TMT mass spectrometry studies above. Batch effect was adjusted using bootstrap regression which modelled genotype, age, sex and batch, but covariance with batch only was removed.

For manuscripts utilizing custom algorithms or software that are central to the research but not yet described in published literature, software must be made available to editors and reviewers. We strongly encourage code deposition in a community repository (e.g. GitHub). See the Nature Portfolio guidelines for submitting code & software for further information.

Data

Policy information about availability of data

All manuscripts must include a data availability statement. This statement should provide the following information, where applicable:

- Accession codes, unique identifiers, or web links for publicly available datasets
- A description of any restrictions on data availability
- For clinical datasets or third party data, please ensure that the statement adheres to our policy

ProteomeXchange title: Native-state proteomics of Parvalbumin interneurons identifies novel molecular signatures and metabolic vulnerabilities to early Alzheimer's disease pathology
ProteomeXchange accession: PXD043963
PubMed ID: 37292756
Publication DOI: Not applicable
Project Webpage: http://www.ebi.ac.uk/pride/archive/projects/PXD043963
FTP Download: https://ftp.pride.ebi.ac.uk/pride/data/archive/2024/03/PXD043963

Camk2a-CIBOP data can be obtained using dataset identifiers PXD027488 and PXD032161. The 2020 mouse Uniprot database (downloaded from https://www.uniprot.org/help/reference_proteome).

Research involving human participants, their data, or biological material

Policy information about studies with human participants or human data. See also policy information about sex, gender (identity/presentation), and sexual orientation and race, ethnicity and racism.

Reporting on sex and gender	Previously published human post-mortem bulk brain proteomic studies from ROSMAPand Banner Sun Health cohorts were used but only analyzed as individuals who were cognitively normal and without pathology (Controls), those with asymptomatic AD (AsymAD), and those with AD, which was based on the data available from the previously published studies.
Reporting on race, ethnicity, or other socially relevant groupings	See above
Population characteristics	See above
Recruitment	Recruitment information was included in the previous publications in which the data was collected (Wingo et al. 2019, Johnson et al. 2020).
Ethics oversight	N/A

Note that full information on the approval of the study protocol must also be provided in the manuscript.

Field-specific reporting

Please select the one below that is the best fit for your research. If you are not sure, read the appropriate sections before making your selection.

- [x] Life sciences
- [ ] Behavioural & social sciences
- [ ] Ecological, evolutionary & environmental sciences

For a reference copy of the document with all sections, see nature.com/documents/nr-reporting-summary-flat.pdf

Life sciences study design

All studies must disclose on these points even when the disclosure is negative.

Sample size	Male and female mice were used for all experiments with data collected from ≥ 3 mice per experimental condition for all experiments. Sample sizes were a priori established to surpass the adequate sample size required by the given statistical method.
Data exclusions	Data exclusions for specific methods (e.g., patch-clamp electrophysiology, individual cells) were made based on established thresholding for acceptable values (e.g., <20 MOhm Ra) in the respective field.
Replication	Mouse models, consistent biological replicates, materials and quantities used, and methodology were all provided in full transparency for ease of replication by others.
Randomization	Samples/organisms were allocated into groups at random, with the exception of genotype.
Blinding	Analysis was conducted by individuals who were blind to information from sample collection until after analysis was complete.

Reporting for specific materials, systems and methods

We require information from authors about some types of materials, experimental systems and methods used in many studies. Here, indicate whether each material, system or method listed is relevant to your study. If you are not sure if a list item applies to your research, read the appropriate section before selecting a response.

Materials & experimental systems

n/a	Involved in the study	
[ ]	[x]	Antibodies
[x]	[ ]	Eukaryotic cell lines
[x]	[ ]	Palaeontology and archaeology
[ ]	[x]	Animals and other organisms
[x]	[ ]	Clinical data
[x]	[ ]	Dual use research of concern
[x]	[ ]	Plants

Methods

n/a	Involved in the study	
[x]	[ ]	ChIP-seq
[ ]	[x]	Flow cytometry
[x]	[ ]	MRI-based neuroimaging

Antibodies

Antibodies used

4G8 Mouse (IHC) 1:2,000 BioLegend 800701 https://www.biolegend.com/nl-nl/products/purified-anti-beta-amyloid-17-24-antibody-11233?GroupID=BLG15648
COX-5 Mouse (IHC) 1:1,000 (Western blot) 1:1,000 Invitrogen 459120 https://www.thermofisher.com/antibody/product/COX5A-Antibody-clone-6E9B12D5-Monoclonal/459120
GFAP Rat (IHC) 1:1.000 Invitrogen 13-0300 https://www.thermofisher.com/antibody/product/GFAP-Antibody-clone-2-2B10-Monoclonal/13-0300
GFP Goat (IHC) 1:2,000 Rockland 600101215 https://www.rockland.com/categories/primary-antibodies/gfp-antibody-600-101-215/
Iba1 rabbit (IHC) 1:500 Abcam ab178846 https://www.abcam.com/products/primary-antibodies/iba1-antibody-epr16588-ab178846.html
LC3 rabbit (Western blot) 1:1,000 Cell Signaling 12741 https://www.cellsignal.com/products/primary-antibodies/lc3a-b-d3u4c-xp-rabbit-mab/12741
NeuN mouse (Flow cytometry) 1:250 Millipore MAB377 https://www.emdmillipore.com/US/en/product/Anti-NeuN-Antibody-clone-A60,MM_NF-MAB377?ReferrerURL=https%3A%2F%2Fwww.google.com%2F
Parvalbumin rabbit (IHC) 1:100 Abcam ab181086 https://www.abcam.com/products/primary-antibodies/parvalbumin-antibody-epr13091-ab181086.html
Parvalbumin gp (IHC) 1:5,000 Synaptic systems 195004 https://sysy.com/product/195004
Parvalbumin Mouse (IHC) 1:2,000 Sigma SAB4200545 https://www.sigmaaldrich.com/US/en/product/sigma/p3088
RFP Rabbit (IHC) 1:2000 (Flow cytometry) 1:250 Rockland 600-401-379 https://www.rockland.com/categories/primary-antibodies/rfp-antibody-pre-adsorbed-600-401-379/
V5 Rabbit (Western blot) 1:500 Abcam ab206566 https://www.abcam.com/products/primary-antibodies/v5-tag-antibody-sv5-p-k-ab206566.html
Wisteria Floribunda Lectin, Biotinylated (IHC) 1:200 Vector Laboratories B-1355 https://vectorlabs.com/products/biotinylated-

wisteria-floribunda-lectin
β-actin Mouse (Western blot) 1:3,000 Santa Cruz sc-47778 https://www.scbt.com/p/beta-actin-antibody-c4?gad_source=1&gclid=CjwKCAiA6KWvBhAREiwAFPZM7hsalwcv66vGhetgxvaVygBYdQ1u64fzGWqiYsmAFWwLY1AZvVTT7hoCDegQAvD_BwE
Anti-Goat Alexa Fluor 488 (IHC) 1:500 Thermofisher scientific A-11055 https://www.thermofisher.com/antibody/product/Donkey-anti-Goat-IgG-H-L-Cross-Adsorbed-Secondary-Antibody-Polyclonal/A-11055
Anti-Guinea Pig Alexa Fluor 488 (IHC) 1:500 (Flow cytometry) 1:500 Thermofisher scientific A11073 https://www.thermofisher.com/antibody/product/Goat-anti-Guinea-Pig-IgG-H-L-Highly-Cross-Adsorbed-Secondary-Antibody-Polyclonal/A-11073
Anti-Guinea Pig Alexa Fluor 647 (IHC) 1:500 Jackson ImmunoResearch 106-605-003 https://www.jacksonimmuno.com/catalog/products/106-605-003
Anti-mouse Alexa Fluor 488 (IHC) 1:500 Thermofisher scientific A32766 https://www.thermofisher.com/antibody/product/Donkey-anti-Mouse-IgG-H-L-Highly-Cross-Adsorbed-Secondary-Antibody-Polyclonal/A32766
Anti-mouse Alexa Fluor 594 (IHC) 1:500 Thermofisher scientific A21203 https://www.thermofisher.com/antibody/product/Donkey-anti-Mouse-IgG-H-L-Highly-Cross-Adsorbed-Secondary-Antibody-Polyclonal/A-21203
Anti-mouse Alexa Fluor 647 (IHC) 1:500 Thermofisher scientific A31571 https://www.thermofisher.com/antibody/product/Donkey-anti-Mouse-IgG-H-L-Highly-Cross-Adsorbed-Secondary-Antibody-Polyclonal/A-31571
Anti-mouse Alexa Fluor Plus800 (Western blot) 1:10000 Thermofisher scientific A32730 https://www.thermofisher.com/antibody/product/Goat-anti-Mouse-IgG-H-L-Highly-Cross-Adsorbed-Secondary-Antibody-Polyclonal/A32730
Anti-Mouse HRP (Western blot) 1:10000 Jackson ImmunoResearch 115-035-003 https://www.jacksonimmuno.com/catalog/products/115-035-003
Anti-rabbbit Alexa Fluor 800 (Western blot) 1:10000 Thermofisher scientific A32735 https://www.thermofisher.com/antibody/product/Goat-anti-Rabbit-IgG-H-L-Highly-Cross-Adsorbed-Secondary-Antibody-Polyclonal/A32735?gclid=CjwKCAiAi6uvBhADEiwAWiyRdj_CLNwKEGQEGwUrg_VlIZrW6EXgUcQ4WwRneGje6vKcB62FrstoMhoCBbkQAvD_BwE&ef_id=CjwKCAiAi6uvBhADEiwAWiyRdj_CLNwKEGQEGwUrg_VlIZrW6EXgUcQ4WwRneGje6vKcB62FrstoMhoCBbkQAvD_BwE:G:s&s_kwcid=AL!3652!3!516608152215!!!g!!!12825517856!122158235235&cid=bid_pca_aus_r01_co_cp1359_pjt0000_bid00000_0se_gaw_dy_pur_con&gad_source=1
Anti-rabbit Alexa Fluor 594 (IHC) 1:500 (Flow cytometry) 1:500 Thermofisher scientific A21207 https://www.thermofisher.com/antibody/product/Donkey-anti-Rabbit-IgG-H-L-Highly-Cross-Adsorbed-Secondary-Antibody-Polyclonal/A-21207
Anti-rabbit Alexa Fluor 647 (IHC) 1:500 Thermofisher scientific A32795 https://www.thermofisher.com/antibody/product/Donkey-anti-Rabbit-IgG-H-L-Highly-Cross-Adsorbed-Secondary-Antibody-Polyclonal/A32795
Anti-rabbit HRP (Western blot) 1:100,000 Jackson ImmunoResearch 111-035-003 https://www.jacksonimmuno.com/catalog/products/111-035-003
Anti-rat Alexa Fluor 488 Thermofisher scientific A48262 https://www.thermofisher.com/antibody/product/Goat-anti-Rat-IgG-H-L-Highly-Cross-Adsorbed-Secondary-Antibody-Polyclonal/A48262
Streptavidin Alexa Fluor 488 (IHC) 1:500 Thermofisher scientific S11223 https://www.thermofisher.com/order/catalog/product/S11223
Streptavidin Alexa Fluor 680 (Western blot) 1:20,000 Thermofisher scientific S32358 https://www.thermofisher.com/order/catalog/product/S32358
Streptavidin DyLight 488 (IHC) 1:500 Thermofisher scientific A21832 https://www.thermofisher.com/proteins/product/Streptavidin-Protein/21832
Streptavidin DyLight 594 (IHC) 1:500 Thermofisher scientific A21842 https://www.thermofisher.com/proteins/product/Streptavidin-Protein/21842

Validation

All antibody used in this study are validated. The link for each antibody are provided above

Animals and other research organisms

Policy information about studies involving animals; ARRIVE guidelines recommended for reporting animal research, and Sex and Gender in Research

Laboratory animals

C57BL/6J (wildtype[WT]) mice (JAX #000664), Rosa26-TurboID (C57BL/6- Gt(ROSA)26Sortml(birA)Srgj/J, JAX #037890)7, Camk2a-Cre-ert2 (B6;129S6-Tg(Camk2acre/ ERT2)1Aibs/J, JAX #012362)119 and 5xFAD (B6.Cg-Tg(APPSwFlLon,PSENl *M146L*L286V)6799Vas/Mmjax, JAX #034848)17,21 mouse lines were used for experiments in this study and genotyping was performed using primers and polymerase chain reaction (PCR) conditions listed on the vendor website (Jackson labs). All animals were maintained on the C57BL6/L background, following at least 10 serial backcrosses if originally derived from a different or mixed background.

Wild animals

The study did not include wild animals.

Reporting on sex

Male and female mice were used for all experiments with data collected from ≥ 3 mice per experimental condition for all experiments. Sex differences were not observed or reported.

Field-collected samples

The study did not involve samples collected in the field.

Ethics oversight

All experiments involving animal
procedures were approved by the Emory University Institutional Animal Care and Use Committee (IACUC, PROTO201700821) and were in accordance with the ARRIVE guidelines.

Note that full information on the approval of the study protocol must also be provided in the manuscript.

Plants

Seed stocks	N/A
Novel plant genotypes	N/A
Authentication	N/A

Flow Cytometry

Plots

Confirm that:

- [ ] The axis labels state the marker and fluorochrome used (e.g. CD4-FITC).
- [ ] The axis scales are clearly visible. Include numbers along axes only for bottom left plot of group (a 'group' is an analysis of identical markers).
- [ ] All plots are contour plots with outliers or pseudocolor plots.
- [ ] A numerical value for number of cells or percentage (with statistics) is provided.

Methodology

Sample preparation	single cell suspension generated using brain tissue
Instrument	BD LSR II flow cytometer
Software	ANALYSIS BY FlowJo v10
Cell population abundance	Neuronaal cells
Gating strategy	Brain tissue> SIngle mononuclear cells (FSC and SSC), > NeuN positive neurons >TdTomato positive cells

- [ ] Tick this box to confirm that a figure exemplifying the gating strategy is provided in the Supplementary Information.
